# Long-term assessment of functional capacity, muscle function, lung function, and quality of life in survivors of ventilator-associated pneumonia

**DOI:** 10.1016/j.heliyon.2023.e23431

**Published:** 2023-12-09

**Authors:** Larissa Mello Dias, Jenipher Angel da Cruz, Agnaldo José Lopes

**Affiliations:** a Rehabilitation Sciences Post-Graduation Programme, Augusto Motta University Centre (UNISUAM), Rio de Janeiro, Brazil; b Faculty Inspirar, Curitiba, Brazil; c Worker's Hospital Complex, Curitiba, Brazil; d Local Development Post-Graduation Programme, Augusto Motta University Centre (UNISUAM), Rio de Janeiro, Brazil

**Keywords:** Mechanical ventilation, Ventilator-associated pneumonia, Exercise, Quality of life, Rehabilitation

## Abstract

**Background and objective:**

The many patients who develop ventilator-associated pneumonia (VAP) have generated numerous VAP survivors who are not followed up in the long term. This study aimed, primarily, to evaluate the long-term functional capacity, as measured using the Glittre-ADL test (TGlittre), of VAP survivors and, secondarily, to calculate the correlations of TGlittre with muscle and lung function.

**Methods:**

This cross-sectional study evaluated 30 VAP survivors 10 months after discharge from the intensive care unit. The participants underwent the following assessments: TGlittre; respiratory muscle strength; handgrip strength (HGS); spirometry; Functional Assessment of Chronic Therapy (FACIT-F); and Short Form-36 (SF-36).

**Results:**

The median TGlittre time was 95 (81–130)% of predicted, and 30 % of the participants performed poorly on TGlittre. One-third of the participants had abnormal spirometry results. TGlittre time was correlated with weight (r_s_ = −0.412, P = 0.023), body index mass (BMI, r_s_ = −0.400, P = 0.029), forced vital capacity (FVC, r_s_ = −0.401, P = 0.030), HGS (r_s_ = −0.571, P = 0.0009), FACIT-F score (r_s_ = −0.405, P = 0.026), and different SF-36 domain scores. Participants who returned to work had a shorter TGlittre time than those who did not (89 (69–104) vs. 129 (102–183)% predicted). Multiple linear regression indicated that FVC and BMI explained 39 % of TGlittre variability.

**Conclusion:**

VAP survivors had suboptimal functional capacity, low lung function, and general fatigue 10 months after discharge. The longer the TGlittre time was, the worse the lung function, muscle function, general fatigue, and quality of life were and the less likely the patient was to have returned to work.

## 1 Introduction

Ventilator-associated pneumonia (VAP) is the most frequent cause of health care-associated infection in intensive care units (ICUs), affecting 5–40 % of patients who remain on invasive mechanical ventilation (IMV) for longer than two days [1]. The estimated incidence varies with the definition, screening methods, patient populations, and country [2]. In the United States of America, the incidence ranges from 1 to 2.5 cases per 1000 days on IMV, while in Brazil and Europe, respective incidences of 16.8 and 18.3 episodes per 1000 days on IMV have been reported [3,4]. VAP has been associated with a greater number of antibiotic prescriptions in the ICU, longer IMV time, difficulty weaning from ventilation, longer hospital stay, and higher health-related costs [5,6]. Although VAP worsens the clinical condition of patients in the short term and increases the hospital mortality rate in the long term [6,7], little is known about its long-term consequences on lung and muscle function [8,9].

Post-VAP pulmonary complications occur in approximately 25 % of survivors, respiratory failure and diaphragmatic weakness being the most frequent [10]. Prolonged use of IMV, the need for neuromuscular blockers (NBs), and a long ICU stay are factors that negatively influence lung function and quality of life (QoL), even 1 year after critical illness [11]. Soon after discharge from the ICU, changes in lung function can be observed, for example, airflow limitations and a reduction in lung volume [10]. Long-term pulmonary function abnormalities have been reported in survivors of acute respiratory distress syndrome (ARDS). Therefore, evaluations during the first 5 years after ARDS are recommended [12]. Despite these changes described in ARDS survivors, no studies have evaluated long-term pulmonary function in post-VAP patients.

Like the deterioration of lung function, loss of muscle mass and strength is a common complication after admission to the ICU and may contribute to decreased functional capacity and limit activities of daily living (ADLs) [13]. Several factors about the ICU stay contribute to muscle dysfunction, including sedation and NBs, nutritional changes, immobility, and inflammation [14,15]. In patients hospitalized for community-acquired pneumonia (CAP), there is evidence of declines in functionality, QoL, and peripheral muscle strength [16], but no studies have evaluated survivors of VAP, especially on long-term muscle function.

Functional status is characterized by an individual's ability to meet his or her basic life needs. It can be impacted by several factors, such as age, obesity, and chronic diseases [17,18]. Several factors compromise functional capacity and QoL after ICU admission, including prolonged VMI, sepsis, immobility, and multiple-organ dysfunction [19]. One study described the outcomes of IMV patients approximately 1.5 years after hospital discharge, observing that their ICU experience impacted their lives in the form of decreased QoL, physical function, and mental function and frequent loss of jobs; however, the authors did not objectively assess functional capacity during exercise [20].

With advances in intensive care, a group of chronically critical patients have emerged who, historically, would not have survived the acute episode [21]. Greater awareness of the impact of critical illness on QoL has led to the need for research focused on overall patient outcomes rather than crude survival. Despite all the technological advances in the field of intensive care in recent decades, VAP survivors are not followed up longitudinally, and little is known about their long-term functionality. In this context, running tests that provide a systemic perspective of functional capacity are essential. The Glittre-ADL test (TGlittre) was recently created to evaluate functionality. This test consists of a circuit of functional activities considered essential in daily life [22]. Because the TGlittre is more appropriate for assessing functional status than other field tests and can model the length of stay required for people in different clinical conditions [16,22], we believe it can reliably assess the long-term changes that occur in survivors of VAP. We hypothesized that VAP survivors 10 months after ICU discharge would suffer low functional exercise capacity due to ongoing damage to muscle strength and lung function. Thus, the first aim of this study was to evaluate the long-term functional capacity, as measured using TGlittre, in VAP survivors. The second aim was to calculate the correlations of TGlittre with muscle function and pulmonary function.

## 2 Methods

### 2.1 Subjects

Conducted between September 2022 and June 2023, this was a cross-sectional observational study of 30 VAP survivors (out of 60 eligible; age ≥18 years) at 10 months after hospital discharge. These patients were admitted to the ICU of Complexo Hospitalar do Trabalhador, Curitiba, Paraná, Brazil, and, after discharge, were contacted by telephone and asked to participate by returning to the hospital for evaluations. Based on preestablished criteria, VAP was defined as pneumonia that developed 48 h after endotracheal intubation [23]. The following exclusion criteria were applied: cardiopulmonary or cerebrovascular diseases; musculoskeletal disorders, motor or neurological sequelae, or other diseases that prevented the performance of TGlittre; and cognitive disorders that prevented the subject from completing the questionnaires and understanding the instructions of the functional tests.

The protocol was approved by the Research Ethics Committee of the Centro Universitário Augusto Motta under protocol number CAAE-60603622.1.0000.5235, and all patients signed an informed consent form.

### 2.2 Measurements

The following variables were considered in relation to the time of VAP: baseline condition for ICU admission; time in ICU; days of sedation; need and duration of NB use; need for tracheostomy; need and time of IMV; time of weaning from IMV; and total hospitalization time.

QoL was assessed using the Short Form-36 (SF-36). This questionnaire assesses QoL in eight domains: four domains (physical functioning, physical role limitations, bodily pain, and general health perceptions) are combined into a physical component score, and four domains (vitality, social functioning, emotional role limitations, and mental health) are combined into a mental component score. The score ranges from 0 to 100, a higher value meaning a better QoL [24].

Overall fatigue was estimated using the Functional Assessment of Chronic Illness Therapy (FACIT-F) scale, which was originally created to measure general fatigue in cancer patients with anemia. The FACIT-F covers physical fatigue, functional fatigue, emotional fatigue, and the social consequences of fatigue. It has 13 items with five Likert responses ranging from “none” to “a lot”. The items are scored from 0 to 4, and the scores are added together, multiplied by 13 and divided by the number of items actually answered. The overall score ranges from 0 to 52, and higher scores reflect less fatigue [25]. A FACIT-F value ≥ 40.1 is considered normal [26].

Respiratory muscle strength was measured using appropriate equipment (VentBras, São Paulo, Brazil). To measure maximal inspiratory pressure (MIP), each participant was instructed to perform a maximum expiration until reaching residual volume and then perform a maximum forced inspiration against an occluded airway. To measure maximal expiratory pressure (MEP), each participant was instructed to perform maximal inspiration until reaching total lung capacity and then to breathe out for 1–3 s with maximum effort against an occluded airway. Five maneuvers for each of MIP and MEP were performed, and the highest value of each was compared to the Brazilian predicted values [27].

Handgrip strength (HGS) was measured using a portable digital handheld dynamometer (Instrutherm, Dm-90, São Paulo, Brazil). The test followed a standardized protocol [28], which recommends the following positioning of the individual: seated, shoulders adducted and neutrally rotated, elbow flexed to 90°, forearm in a neutral position, and wrist between 0 and 30° of dorsiflexion. The dominant hand was used. Each assessment included three attempts, the highest value being recorded in kilograms and compared to the predicted values [29].

Spirometry was performed with dynamic equipment (Minispir Waukesha, WI, USA) following American Thoracic Society/European Respiratory Society recommendations [30]. The following parameters were evaluated: forced vital capacity (FVC), forced expiratory volume in 1 s (FEV_1_), and the FEV_1_/FVC ratio. The reference values were obtained using equations for the Brazilian population developed by Pereira et al. [31], and the results are expressed as percentages of the predicted values (% predicted).

Functional capacity was assessed using TGlittre as originally proposed [22]. Briefly, the test consists of completing a 10-m circuit in which each person starts in a sitting position and then gets up, walks, goes up and down two steps, and walks to a bookshelf with two shelves whose height is adjustable for each participant. Next, the person must move three 1-kg objects, one by one, from the top shelf to the bottom shelf, then to the floor, back to the bottom shelf, and finally back to the top shelf. After this activity, the person walks the course in the opposite direction, constituting one lap. The test is complete after the person finishes five laps. For the analysis of TGlittre, the best time out of two runs was recorded, and the result was compared with the Brazilian reference values [18].

### 2.3 Statistical analysis

Statistical analysis was performed with SPSS version 26. The Gaussian distribution of the data was tested using the Shapiro–Wilk test and the graphical analysis of histograms. Descriptive data are expressed as measures of central tendency and dispersion suitable for numerical data, and categorical data are presented as frequencies and percentages. The relationship between the TGlittre time (% predicted) and different numerical variables was analyzed with Spearman correlation coefficients, and the categorical variables were analyzed using the Mann–Whitney test. Multivariate analysis using stepwise forward selection at the 5 % level was performed using multiple linear regression, which identified the independent variables that explained the logarithmic variability in TGlittre time (% predicted). This analysis was applied with natural logarithmic transformation [ln(TGlittre time % predicted)], aiming to adapt its distribution to a parametric approach. The level of significance was 5 %.

## 3 Results

During hospitalization in the ICU, 216 patients developed VAP. Of these, 127 died, 2 of whom died after discharge. Of the 89 survivors, 19 were excluded for the following reasons: severe traumatic brain injury (n = 17), spinal cord injury (n = 7), and cognitive deficits (n = 5). Of the 60 eligible for the study, 35 were contacted, and 30 came in for the evaluations. The sample evaluated was mostly adult men. At the time of VAP, the main underlying conditions were traumatic brain injury and orthopedic surgery, followed by COVID-19 pneumonia and postoperative thoracoabdominal surgery. The sociodemographic parameters and clinical data at the time of VAP are shown in Table 1.

**Table 1:** Sociodemographic parameters and clinical data at the time of ventilator-associated pneumonia.

Variable	Values
Sociodemographic	
Male/female	25/5
Age (years)	36.2 ± 16.2
Weight (kg)	75.1 ± 18.4
Height (m)	1.73 ± 0.10
BMI (kg/m^2^)	25 ± 5.2
**Cause of ICU admission**	
Traumatic brain injury	13 (43.3 %)
Orthopedic surgery	9 (30 %)
COVID-19 pneumonia	6 (20 %)
Thoracoabdominal surgery	2 (6.7 %)
**Hospitalization information**	
Total length of stay (days)	37 (23–51)
Time in ICU (days)	24 (17–38)
Sedation time (days)	7.5 (4–13)
Need for NB	
Yes	13 (43.3 %)
No	17 (56.7 %)
Time of use of NB (days)	0 (0–5.3)
Need for tracheostomy	
Yes	21 (70 %)
No	9 (30 %)
IMV time (days)	16 (10–27)
IMV weaning time (days)	9 (4.8–15)

Values are the mean ± SD, median (interquartile range), or number (%). BMI: body mass index; ICU: intensive care unit; NB: neuromuscular blocker; IMV: invasive mechanical ventilation.

The median time after VAP was 295 (270–336) days. In the spirometry test, 20 (66.7 %), 5 (16.7 %), 3 (10 %), and 2 (6.7 %) participants had a normal pattern, restrictive pattern, obstructive pattern, and mixed pattern, respectively. Eight (26.7 %) participants had a FACIT-F score <40.1, indicating general fatigue. Regarding employability, 20 (66.7 %) participants had already returned to work, and 10 (33.3 %) had not yet re-entered the labor market. The SF-36 domains with the lowest scores were emotional role limitations, general health perceptions, and mental health. The pulmonary function, muscle strength, general fatigue, and QoL values are given in Table 2.

**Table 2:** Pulmonary function, muscle strength, general fatigue, and quality of life in the studied sample.

Variable	Values
Pulmonary function	
FVC (% predicted)	87 (80–100)
FEV_1_ (% predicted)	92 (81–101)
FEV_1_/FVC (%)	83 (78–92)
**Muscle strength**	
MIP (% predicted)	82 (53–108)
MEP (% predicted)	87 (60–105)
HGS (% predicted)	93 (73–118)
**General fatigue**	
FACIT-F (points)	44 (38–49)
**Short Form-36 (points)**	
Physical functioning	75 (54–91)
Physical role limitations	75 (25–100)
Bodily pain	72 (52–84)
General health perceptions	64 (53–72)
Vitality	73 (55–86)
Social functioning	75 (50–100)
Emotional role limitations	33 (0–100)
Mental health	70 (48–80)

Values are mean ± SD or median (interquartile range). FVC: forced vital capacity; FEV_1_: forced expiratory volume in 1 s; MIP: maximum inspiratory pressure; MEP: maximum expiratory pressure; HGS: handgrip strength; FACIT-F: Functional Assessment of Chronic Illness Therapy.

**Fig. 1:**
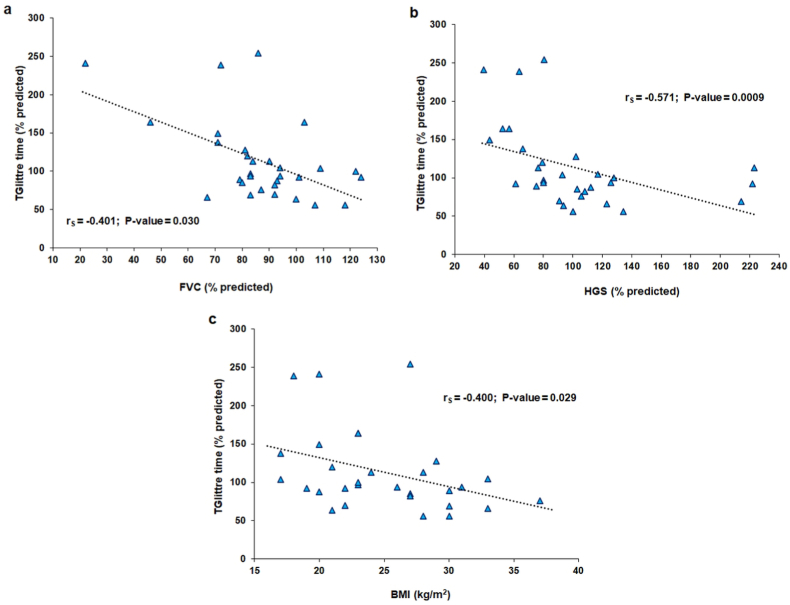
Relationships of Glittre-ADL test (TGlittre) time with body mass index (BMI) **(a)**, forced vital capacity (FVC) **(b)**, and handgrip strength (HGS) **(c)** in survivors of ventilator-associated pneumonia.

**Fig. 2:**
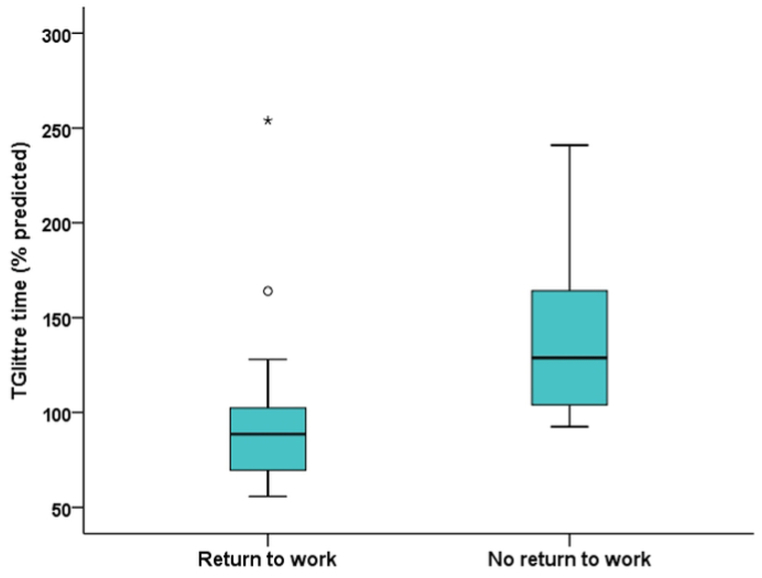
Comparison of Glittre-ADL test (TGlittre) time between survivors who returned to work and those who did not.

The median TGlittre time was 2.29 (1.97–3.10) min. When the values were compared to the Brazilian predicted values reported by Reis et al. [18], the median for the participants was 95 (81–130) % predicted, and only 9 (30 %) participants had a TGlittre time >120 % predicted. No patient exhibited desaturation (≥4 % decrease) during TGlittre. TGlittre time was significantly correlated with several parameters, including body index mass (BMI), FVC, and HGS (Fig. 1). TGlittle time was also correlated with weight, FACIT-F score, and several domains of SF-36. The correlation coefficients between TGlittre time and sociodemographic parameters, clinical data at the time of hospitalization for VAP, pulmonary function, muscle strength, general fatigue, and QoL are given in Table 3. Regarding the categorical variables, participants who returned to work had shorter TGlittre time that was shorter than the TGlittre time for those who had not yet returned to work (89 (69–104) vs. (129 (102–183) % predicted) (Fig. 2). There were no significant differences in baseline condition for admission to the ICU, need for NB use, or need for tracheostomy.

**Table 3:** Spearman's correlation coefficients for the Glittre-ADL test, sociodemographic parameters, clinical data at the time of ventilator-associated pneumonia, lung function, muscle strength, general fatigue, and quality of life.

Variable	TGlittre time	
	r_s_	P value
Sociodemographic		
Age	0.019	0.92
Weight	−0.412	**0.023**
Height	−0.195	0.30
**Data obtained at the time of VAP**		
Total length of stay	0.103	0.59
Time in ICU	−0.010	0.96
Sedation time	−0.233	0.22
Time of use of NB	0.002	0.99
IMV time	−0.011	0.96
IMV weaning time	−0.041	0.83
**Data obtained after 10 months of VAP**		
FEV_1_ (% predicted)	−0.314	0.09
FEV_1_/FVC (%)	0.074	0.70
MIP (% predicted)	−0.164	0.39
MEP (% predicted)	−0.207	0.27
FACIT-F (points)	−0.405	**0.026**
Physical functioning (points)	−0.375	**0.041**
Physical role limitations (points)	−0.564	**0.001**
Bodily pain (points)	0.019	0.92
General health perceptions (points)	−0.251	0.18
Vitality (points)	−0.434	**0.016**
Social functioning (points)	−0.477	**0.007**
Emotional role limitations (points)	−0.437	**0.015**
Mental health (points)	−0.234	0.21

BMI: body mass index; VAP: ventilator-associated pneumonia; ICU: intensive care unit; NB: neuromuscular blocker; IMV: invasive mechanical ventilation; FVC: forced vital capacity; FEV_1_: forced expiratory volume in 1 s; MIP: maximum inspiratory pressure; MEP: maximum expiratory pressure; HGS: handgrip strength; FACIT-F: Functional Assessment of Chronic Illness Therapy.

In the multiple linear regression, FVC (P = 0.001) and BMI (P = 0.011) were the only independent variables that predicted TGlittre time. The overall explanatory power of the model was good (R^2^ = 0.39).

## 4 Discussion

In this study, which is one of the few that has addressed the long-term outcomes of patients who needed ICU admission, the main findings were that 10 months after VAP, survivors had TGlittre times that were close to normal, but one-third of the sample had abnormal spirometry patterns. Although fewer than one-third of VAP survivors had general fatigue, two-thirds had already returned to work. There were associations between TGlittre time and pulmonary function, muscle function, general fatigue, QoL, and return to work. The only variables that explained TGlittre time were FVC and BMI. To the best of our knowledge, this is the first study to longitudinally evaluate VAP survivors using TGlittre.

A long ICU stay can reduce functional capacity during exercise due to persistent weakness and can also lead to a spectrum of long-term physical and neuropsychological impairments [12]. Although 30 % of our sample showed poor on TGlittre, performance was close to normal for most survivors. Our findings are in agreement with the results reported by Herridge et al. [12], who found a median distance walked in the 6-min walk test (6MWT) of 76 % of that predicted for survivors of stroke and ARDS at 5 years after ICU discharge. These authors observed that the physical functional recovery rate was higher for younger patients than for older patients. In contrast to these two studies, José and Corso [16] observed worse performance in the 6MWT and the TGlittre when patients hospitalized for CAP were compared to controls. They also showed worse muscle strength. This indicates that in addition to the severity of the clinical condition, it is important to consider the time after pneumonia and/or ICU stay. Another factor that explains the good performance of our patients is their relatively young mean age (36.1 ± 16.2 years), possibly influenced by the number of individuals with VAP after COVID-19, which may have impacted the recovery of functionality.

In this study, pulmonary function was normal in two-thirds of the patients, a finding that is consistent with the results of the study by Herridge et al. [12] of ARDS survivors evaluated after 5 years (pneumonia and sepsis were the most common risk factors), in whom pulmonary function was normal or nearly normal. Different results were observed by Abentroth et al. [10], who evaluated patients who had been admitted to the ICU (78 % after surgery and trauma) 3 months after hospital discharge. In their sample, 73 % of the patients showed pulmonary dysfunction on spirometry. These authors observed that patients with greater functional dependence had lower FVC and FEV_1_ values, were older, spent more days on IMV, and had longer ICU stays. These findings support the hypothesis that the time of post-ICU evaluation is a key variable in predicting patient outcomes. Notably, in their multivariate analysis, FVC was the main variable that explained TGlittre time, indicating the importance of evaluating the contribution of respiratory mechanic disorders to functional capacity, as impaired lung function contributes to exercise intolerance and impairs the performance of ADLs [10]. Although we only investigated VAP, true lung parenchymal morbidity in ARDS survivors is uncommon, and when present, persistent restrictive disease is likely related to diaphragmatic weakness and mild D_LCO_ impairment [9].

In critically ill patients, including those with VAP, more than 20 % of total muscle mass may be lost by the end of the first week in the ICU, muscle loss being greater in those with worsening organ failure, making it important to longitudinally evaluate muscle mass and muscle function [21]. In this study, HGS was relatively preserved 10 months after VAP, although general fatigue, as assessed using the FACIT-F, was detected in almost one-third of our patients. HGS is less influenced by the degree of physical activity than leg muscle strength is and therefore is more suitable for assessing and predicting the development of functional disabilities and outcomes related to longevity and mortality [32]. Notably, HGS was correlated with TGlittre time (r = −0.571, P = 0.0009). In fact, higher HGS values have been associated with better lung function and better performance in mobility tests in patients after pneumonia [16,32]. José and Corso [16] observed that like patients with VAP, post-CAP patients often experienced fatigue. General fatigue, when associated with more intense inflammatory processes and/or the use of corticosteroids in patients who have been hospitalized for a long time, can affect lower tolerance and generate more severe muscle dysfunction [16].

Like muscle strength, QoL in VAP survivors should be performed as early as possible after the ICU stay and followed longitudinally to assess outcomes and outline rehabilitative strategies [33]. In this study, there was significant impairment of QoL, especially in the domains that compose the mental component of the SF-36, and interestingly, there were significant correlations between TGlittre time and several of the domains of the SF-36. Evaluating the long-term outcomes of 130 patients undergoing IMV, Li et al. [20] observed that experience in the ICU had negative impacts, worsening both QoL and professional issues. Unlike us, those authors observed greater impairment in the physical component domains of the SF-36, a result that can be explained at least in part by the higher mean age of their sample (60.1 ± 15.4 years) and the fact that only 23 % of the patients had a diagnosis of VAP. Despite the negative impact of VAP, two-thirds of our patients had already returned to work. Studying patients with ARDS 1 year after hospital discharge, McHugh et al. [34] found that only 25 % of survivors had returned to work, indicating a more negative impact of ARDS compared to that of VAP on long-term employability.

This study has some limitations. First, the sample was relatively small. The search for VAP survivors is hampered by the lack of regular follow-up. Second, because there was no initial assessment of functional capacity, we do not know whether these changes were due to previous conditions or secondary to the acute disease or the need for VAP. However, this limitation applies to almost all studies analyzing ICU survivors. Third, we did not have the patients' premorbid lung function results. If we consider we had no patients with chronic lung diseases and that almost 70 % of the sample had normal spirometric parameters 10 months after discharge, we can assumed that these patients had normal lung function before admission to the ICU. Fourth, some parameters that could affect the functional outcome were not evaluated, such as the ratio of arterial oxygen partial pressure to fractional inspired oxygen, level of positive end-expiratory pressure, use of steroids and/or vasopressors, and presence of septic shock. This is a difficulty in studies that involve retrospective data collection, although we think that the time when the assessments were carried out was so long after hospitalization that these factors could hardly have impacted functional exercise capacity. Finally, we did not objectively measure the physical activity of patients after ICU discharge with an activity monitor, nor did we measure their inflammatory status, which could have helped explain our findings. Despite these limitations, our findings serve as a starting point for follow-up tests in this population of patients, including the validation of the use of TGlittre in the determination of variations in functional capacity during exercise.

## 5 Conclusion

About one-third of our VAP survivors had low functional capacity as assessed using TGlittre, low lung function, and general fatigue 10 months after discharge. The longer the TGlittre time was, the worse the lung function, muscle function, general fatigue, and QoL, and the less likely the individual was to have returned to work. FVC and BMI were the predictive variables of TGlittre time in this population. After hospital discharge, many patients are lost to follow-up, and due to the lack of resources and medications, their health status may deteriorate. Therefore, rehabilitative strategies to minimize peripheral muscle and functional impairment should be offered to VAP survivors, as multidisciplinary programs for the most vulnerable and dependent patients can generate satisfactory results.

## Ethics approval and consent to participate

This study was approved by the Research Ethics Committee of the Centro Universitário Augusto Motta under protocol number CAAE-60603622.1.0000.5235. All participants signed an informed consent form.

## Consent for publication

Not applicable.

## Funding

The authors wish to thank the Conselho Nacional de Desenvolvimento Científico e Tecnólogico [CNPq; Grant number #301967/2022-9), Brazil, the Fundação Carlos Chagas Filho de Amparo à Pesquisa do Estado do Rio de Janeiro (FAPERJ; Grant numbers #E−26/010.002124/2019, #E−26/211.187/2021, #E−26/211.104/2021, and #E−26/200.929/2022), Brazil, and the Coordenação de Aperfeiçoamento de Pessoal de Nível Superior (CAPES, Finance Code 001, 88881.708719/2022-01, and 88887.708718/2022-00), Brazil.

## Data availability statement

Data will be made available on request.

## Author agreement/declaration

All authors reviewed and approved the final form of the submitted paper. The authors declare that this work is original and that all information, including all tables and figures, was generated by the authors, has not been published, and is not under consideration elsewhere.

## CRediT authorship contribution statement

**Larissa Mello Dias:** Writing – review & editing, Writing – original draft, Validation, Project administration, Formal analysis, Conceptualization. **Jenipher Angel da Cruz:** Writing – review & editing, Writing – original draft, Investigation, Data curation. **Agnaldo José Lopes:** Writing – review & editing, Writing – original draft, Validation, Supervision, Funding acquisition, Formal analysis, Conceptualization.

## Declaration of competing interest

The authors declare that they have no known competing financial interests or personal relationships that could have appeared to influence the work reported in this paper.
